# Consumer Preferences, Perceived Benefits, and Determinants of Fruit and Vegetable Consumption in Urban Kumasi, Ghana

**DOI:** 10.1002/fsn3.71592

**Published:** 2026-04-28

**Authors:** Stephen Opoku‐Mensah, Collins Yeboah Asiedu, Benjamin Sarfo

**Affiliations:** ^1^ Department of Agropreneurship, Faculty of Entrepreneurship and Enterprise Development Kumasi Technical University Kumasi Ghana; ^2^ Cocoa Health and Extension Division Ghana Cocoa Board Ajumako Ghana; ^3^ Department of Agricultural Economics and Extension Education Akenten Appiah Menka University of Skills Training and Entrepreneurial Development Mampong Ghana

**Keywords:** consumption behavior, fruit and vegetable, negative binomial regression, perceived benefits, sensory attributes, urban consumers

## Abstract

This study examined fruit and vegetable (FV) consumption patterns within the context of Ghana's transforming food systems, driven by urbanization, economic growth, and evolving consumer preferences. Primary data was sourced from 150 respondents across low‐, middle‐, and high‐income communities in urban Kumasi. Descriptive statistics, Kendall's coefficient of concordance, and Negative Binomial Regression were employed to analyze the data. The results showed that consumers prioritized intrinsic attributes (taste, freshness, and appearance), extrinsic attributes (price and nutritional value), and environmental‐related attributes (organic and the presence of pesticides). Though the majority of respondents have a positive perception of the health and nutritional benefits of FV consumption, the actual daily intake is still abysmally low, demonstrating the gap between knowledge and practice. Factors that significantly influence FV consumption are the number of dependents, accessibility, social and cultural influences, distance to markets, and nutrition knowledge. Surprisingly, refrigerator ownership and shared household decision‐making were negatively associated with vegetable intake, possibly reflecting behavioral substitutions for non‐perishable produce. These findings show that behavioral and contextual factors, rather than economic attributes, serve as the primary determinants of urban dietary behavior. This paper recommends key stakeholders to pursue awareness campaigns for consumers to incorporate FV as part of daily dietary habits as part of overall wellbeing. In addition, the main agents across the urban FV supply chain (informal vendors and retailers) should be encouraged to capitalize on consumers' preference for sensory qualities such as taste, appearance, freshness, price, nutrition, organics, and seasonality, to meet demands and boost consumption.

## Graphical abstract

Fruits and vegetables (FVs) are essential for meeting daily dietary requirements for good health and quality of life. Food systems transformation on account of increasing population, urbanization, and improved living standards in emerging economies are driving dietary consumer taste and preference among urban dwellers. This study examined fruit and vegetable (FV) consumption patterns using primary data from 150 respondents in urban Kumasi, Ghana. The results showed that consumers prioritized intrinsic attributes (taste, freshness, and appearance), extrinsic attributes (price and nutritional value), and environmental related attributes (organic and pesticides‐free FV). While respondents have a positive perception of the health and nutritional benefits of FV consumption, the actual daily intake is still abysmally low. FV consumption was influenced by the number of dependents in the household, accessibility, social and cultural influences, distance to markets, and nutrition knowledge. These findings show that behavioral and contextual factors, rather than economic attributes, serve as the primary determinants of urban dietary behavior.

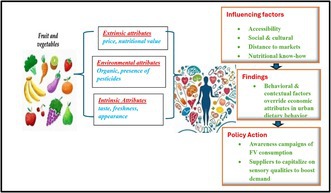

## 1 Introduction

Urbanization is transforming food systems worldwide, particularly in low‐ and middle‐income countries (LMICs), where rapid demographic shifts and changing consumption patterns are reshaping diets and public health outcomes (Baker et al. 2020; Reardon et al. 2020). By 2050, 68% of the global population is expected to live in urban areas, with sub‐Saharan Africa (SSA) experiencing the fastest urban growth at an annual rate of 4.1% (UN‐DESA 2019; McDermott and Wyatt 2017). These transformations come with significant shifts in the dietary habits and consumption patterns of urban dwellers, sometimes with detrimental effects. For example, studies show that an increased consumption of energy‐dense, nutrient‐poor foods is contributing to a dual burden of undernutrition and non‐communicable diseases (NCDs) such as diabetes and cardiovascular conditions (Popkin and Ng 2022; Hawkes et al. 2020). In most of SSA, NCDs accounted for 37% of total deaths in 2019, up from 24% in 2000, highlighting the urgent need to promote more healthy and nutritious diets, particularly fruits and vegetables (FVs) (Juma et al. 2020; Afshin et al. 2019). Despite the World Health Organization's (WHO) recommendation of a minimum daily intake of 400 g of fruits and vegetables (FVs), per capita consumption in SSA remains critically low at 268 g (Mensah et al. 2021; Beal et al. 2017).

These global disparities are particularly evident in Ghana, where urbanization and dietary shifts intersect with unique socioeconomic realities. Data from the Ghana Statistical Service show that urban households consume 74 kg of FVs annually, surpassing rural consumption but still falling short of WHO recommendations (Ghana Statistical Service 2021). Rapid urbanization, particularly in mid‐sized urban centers across SSA, has populations with diverse food systems that are heavily reliant on informal markets (Abdulai et al. 2017). Informal vendors supply approximately 80% of the Kumasi FVs (Kushitor 2023), with consumer choices influenced by affordability, freshness, and trust in vendor quality. However, FV consumption remains uneven across residential income groups due to price sensitivity, seasonal availability, and a growing dependence on processed foods (Janice et al. 2024; Nsiah‐Asamoah and Amoah 2018; Awuni et al. 2017).

While households in Kumasi epitomize the practical implications of these dynamics, existing literature reveals important gaps in how FV consumption patterns are studied in urban settings in Ghana. Over the past decade, several studies have examined Ghana's food systems and dietary transitions, often concentrating on supply side factors such as pesticide residues (Agyapong et al. 2022; Kushitor 2023; Oppong‐Kyeremeh and Bannor 2021), pathogen contamination (Dapaah Opoku et al. 2020; de Jager et al. 2018), ecological variation (Andam et al. 2018; Van Asselt et al. 2018; Ghose and Yaya 2018), rural food insecurity (Meng et al. 2018; Awuni et al. 2017; Frempong and Annim 2017) and general dietary trends (Chagomoka et al. 2015; Florkowski et al. 2012; Bempah and Donkor 2011). However, empirical research on demand‐side dynamics, focusing on consumer preferences, perceptions, and purchasing behaviors remains limited. Few studies have examined in‐depth urban consumer behavior, and city‐specific differences in FV access and its consumption patterns by type, frequency, or retail structure, especially in secondary cities (Faye et al. 2024; Mergenthaler et al. 2009). What comes closer is a study by Wongnaa et al. (2024), that focused on organic vegetable consumption patterns and determinants in Urban Ghana.

This study examines consumers operating within informal food environments and addresses these empirical gaps by investigating fruit and vegetable (FV) consumption in urban Kumasi from a demand‐side perspective. The objectives of this paper are to: (i) identify the preferred attributes of fruits and vegetables that shape urban purchasing decisions, (ii) assess consumer‐perceived benefits of FV consumption, (iii) examine urban consumption patterns, and (iv) analyze the key factors influencing these patterns. In terms of practical relevance, this study provides context‐specific insights for urban food policymakers, municipal planners, and stakeholders working to enhance nutrition‐sensitive governance in Ghana's rapidly urbanizing cities. This study contributes to the limited empirical literature on the demand‐side dynamics of fruit and vegetable consumption in West African urban environments. Findings from the study are expected to contribute to shaping the national priorities toward nutrition‐sensitive food systems, particularly those aligned with Sustainable Development Goals (SDG) 2.1 (ensuring access to safe, nutritious food) and SDG 3.4 (reducing premature mortality from non‐communicable diseases by 2030).

## 2 Literature Review

The transformation of food systems in low‐ and middle‐income countries (LMICs) has accelerated in recent decades, driven by rapid urbanization, demographic changes, and shifting dietary patterns (Afshin et al. 2019).

The health benefits associated with FV consumption, including their role in preventing chronic diseases and promoting overall well‐being (Okyere et al. 2024), shape dietary choices in urban Ghana. While urban consumers increasingly recognize these benefits (Awuni et al. 2017), economic constraints often prevent the translation of knowledge or awareness into practice (Dijkxhoorn et al. 2021; Seidu et al. 2021). The deep cultural embedding of FVs in traditional Ghanaian meals and communal practices enhances their perceived value beyond nutritional considerations (Janice et al. 2024; Oppong‐Kyeremeh and Bannor 2021), potentially explaining the continued emphasis on sensory attributes like freshness and visual appeal that consumers associate with both health and cultural authenticity (Florkowski et al. 2012).

While urban residents typically enjoy greater physical access to food outlets, they are often offset by affordability constraints, supply instability, and food safety concerns (Fanzo 2023; Fanzo et al. 2020). These unique challenges have been echoed by Akparibo et al. (2021) including the dominance of informal markets affecting urban consumption behavior.

### 2.1 Factors Influencing Fruit and Vegetable Consumption

Fruit and vegetable consumption patterns and the choices made by consumers are influenced broadly by several factors, ranging from household and individual socioeconomic factors to sensory, environmental, and a combination of other factors. The influence of socio‐economic variables on FV choices tends to be mixed depending on the specific geographical location, cultural settings, and other such factors. Key individual‐level factors include demographic variables such as age, gender, education, and income (Odum et al. 2018; Amo‐Adjei and Kumi‐Kyereme 2015; Meng et al. 2014; Doku et al. 2013), along with psychosocial factors like nutrition knowledge, self‐efficacy, and attitudes (Fayasari et al. 2020).

Socioeconomic attributes status remains a particularly strong predictor of consumption patterns (Kushitor et al. 2023; Ebadi and Ahmadi 2019). Evidence from the literature indicates that females tend to consume higher and more frequent FV than males (Hall et al. 2022; Pengpid and Peltzer 2018; Mayén et al. 2016). Educational attainment positively correlates with FV consumption (Stadlmayr et al. 2023; Oppong‐Kyeremeh and Bannor 2021). Most studies show that there is a positive relationship between educational status and FV consumption for both genders. Similarly, FV consumption has been shown to be directly related to age, as evidence from the literature shows that older people consume more FV than younger ones (Hall et al. 2022; Sinyolo et al. 2020). In terms of occupation, the type of job of consumers also influences FV choice, however, there appears to be no specific patterns regarding types of education and choice of FV by consumers (Amo‐Adjei and Kumi‐Kyereme 2015; Lomira et al. 2021; Wang et al. 2019).

A large body of literature supports the view that sensory attributes, made up of intrinsic and extrinsic factors, have a profound influence on consumers' choice and preference for food products such as FVs. Food intrinsic factors constitute the inherent physical attributes contained in food products such as taste, color, smell, aroma, texture, brightness, freshness, crunchiness (Ahmed and Basu 2016; Petrescu et al. 2017). They are perhaps the very first set of attributes that attract and draw customer attention. Extrinsic food attributes that influence consumer choice and preference include the price, promotional offers, expiry dates, country of origin, labelling, and brand information (Wang et al. 2019; Alfonzo et al. 2018; Tsironi et al. 2017; Fandos and Flavian 2006). Other significant factors closely related to sensory attributes that influence food choices include food safety perceptions (Dzudzor et al. 2024; Amfo et al. 2019), cultural and personal preferences (Nyarko and Bartelmeß 2024; Kähkönen et al. 2021), and exposure to nutrition education programs (Saha et al. 2020). Additionally, health consciousness, perceived quality, and trust in food sources play particularly important roles in contexts where food safety concerns are prevalent (Kumar et al. 2025; Adams et al. 2018), with growing consumer interest in organic FV options perceived as safer and more nutritious (Wongnaa et al. 2024; Nsiah‐Asamoah and Amoah 2018). Another pertinent factor that invariably drives consumer choices is price and income.

Conventionally, price is considered a barrier to the purchasing decision of consumers of high value commodities like FVs (Mayén et al. 2016; Nguyen et al. 2021; Karelakis et al. 2019). Thus, high commodity prices can potentially deter consumer demand for FVs. Similarly, income emerges as a key determinant of FV consumption (Frank et al. 2019; Miller et al. 2016). Higher‐income households not only consume more vegetables (Mensah et al. 2021) but also prioritize health‐driven purchases (Meng et al. 2018), while low‐income urban populations face persistent affordability barriers (Dijkxhoorn et al. 2021; Miller et al. 2016). This income‐consumption relationship is further mediated by location‐specific factors (Meng et al. 2014), with the positive correlation between income and FVs expenditure well‐documented (Meng et al. 2018).

Environmental and community‐level factors including market proximity, neighborhood characteristics, and infrastructure availability (e.g., refrigeration access) collectively shape urban FV consumption patterns. Physical access to food outlets combined with information channels creates important synergies, as demonstrated in South Africa where both proximities to markets and media exposure positively influenced intake (Sinyolo et al. 2020). The urban food environment exerts substantial influence on dietary patterns (Mockshell et al. 2022), with grocery stores and farmers' markets promoting healthier consumption (Gustafson et al. 2013) while areas dominated by fast‐food outlets typically show reduced FV intake. These environmental factors disproportionately disfavor low‐income communities where limited FV availability exacerbates nutritional disparities (Kushitor et al. 2023).

Despite this growing evidence, significant gaps remain in understanding Ghana's urban FV consumption dynamics. Most existing research focuses on production and supply‐side factors like fruit and vegetable crop production and output, and pesticide contamination or broad dietary trends (Agyapong et al. 2022; Meng et al. 2018; Chagomoka et al. 2015; Bempah and Donkor 2011), with relatively limited attention to consumer‐side behaviors (Amo‐Adjei and Kumi‐Kyereme 2015). Second, the common practice of aggregating fruits and vegetables into a single category obscures important differences in their consumption patterns (Kpodo et al. 2015). In addition, the research focus on Ghana's capital city, Accra, has left secondary cities like Kumasi with their distinct retail ecosystems largely underrepresented (Tay and Ocansey 2022; Florkowski et al. 2012). This lack of city‐specific evidence risks creating policy misalignments with the realities of secondary urban centers where informal markets and socio‐cultural norms strongly influence consumption behaviors.

### 2.2 Theoretical Framework: Theory of Planned Behavior (TPB)

The Theory of Planned Behavior (TPB), developed by Ajzen (1991), has been widely applied to explain and predict human decision‐making in health, nutrition, and food consumption studies. It extends the Theory of Reasoned Action (Ajzen and Fishbein 1980) by incorporating perceived behavioral control, thus recognizing that human behavior is shaped not only by intention but also by the perceived ease or difficulty of performing the behavior. Numerous studies in nutrition and public health have used TPB to examine fruit and vegetable consumption behaviors (O'Neal et al. 2012; Kothe et al. 2012; Povey et al. 2000).

According to the TPB, behavioral intention is determined by three key components: attitude, subjective norms, and perceived behavioral control. In this study, attitude refers to an individual's evaluation of fruit and vegetable consumption as beneficial based on their nutritional knowledge and perceived health benefits. Prior studies, such as those by Kothe et al. (2012) and Povey et al. (2000), have shown that positive attitudes toward the nutritional value of fruits and vegetables significantly increase both intention and actual consumption. Subjective norms represent perceived social pressures or expectations from significant others that influence dietary behaviors. Within urban Ghanaian households, these social influences are often expressed through shared food decision‐making and participation in cultural or community‐based events that promote or discourage fruit and vegetable intake. This is consistent with the findings of O'Neal et al. (2012), who observed that social norms strongly predict dietary choices in collectivist societies.

Perceived behavioral control reflects an individual's perception of factors that facilitate or hinder fruit and vegetable consumption. In this study, accessibility, distance to markets, income level, and refrigerator ownership were used as indicators of this construct. Households that perceive fruits and vegetables as easily accessible or storable are more likely to consume them regularly. Empirical evidence from Heard et al. (2020) in Vietnam and Zheng et al. (2025) in China supports the idea that resource availability and infrastructure shape dietary patterns by influencing convenience and preservation. In addition, demographic factors such as age, gender, education, and marital status indirectly influence these three constructs by shaping attitudes, social expectations, and perceived control. As highlighted by Povey et al. (2000), these demographic characteristics act as moderators that influence how intention translates into actual behavior of consumers. Hence, in this study, the Theory of Planned Behavior (TPB) was employed as a theoretical framework to interpret how attitudes, social influences, and perceived control collectively shape household decisions and actual consumption of fruits and vegetables in urban Kumasi.

## 3 Material and Methods

### 3.1 Study Area

The study was conducted in Kumasi Metropolis, the second largest city in Ghana, the capital of the Ashanti Region of Ghana. The study area falls within latitudes 6.35°N and 6.40°S and longitudes 1.30°W and 1.35°E and has a population of 1,379,335 (GSS 2021). It serves as an important center for commercial activities and a migration hub with 413,561 households, an average of three persons per household (GSS 2021). The city contains 8.2% of the Ashanti Region's total population, with 71.4% of the population residing in the informal sector (GSS 2021). The region has a 61% urban population, which supersedes the national average of 59.24% (World Bank 2023).

### 3.2 Research Design, Sample Size, Sampling Technique and Data Collection

This study employed a quantitative approach to examine fruit and vegetable consumption patterns among household in the Kumasi Metropolis. Primary data were collected using structured questionnaires administered across low‐, middle‐, and high‐income communities, stratified according to the established classifications of the Kumasi Metropolitan Assembly (KMA) (Nimoh et al. 2018). The focus was on individuals in households since most working‐class urban dwellers may make independent choices in the consumption of F.V.

The sample size was determined using Cochran's formula for large populations (Cochran 2009), which is suitable when the total population is either undefined or sufficiently large such that the sample represents < 5% of the total population. Assuming a 95% confidence level $\left(Z=1.96\right)$, a 50% response distribution $\left(p=0.5\right)$, and an 8% margin of error $\left(e=0.08\right)$, the sample size was calculated using the following equation:

(1) $$n=\frac{Z^{2}.p.q}{e^{2}}$$

where q=1−p. Applying the values yields a final sample size of 150, as shown in Equation (2).

(2) $$n=\frac{1.96^{2}\times 0.5\times 0.5}{0.08^{2}}=150$$

In Ghana, studies have consistently indicated that the intake of fruits and vegetables is not up to the recommended levels among the population groups. For instance, about 28% of women have a daily fruit intake as recommended, about 27% of adolescents have an adequate vegetable intake, and nearly half of urban adults eat fruits and vegetables regularly but are still below the recommended five daily servings (Kubuga and Aguree 2025; Seidu et al. 2021; Tachi et al. 2020; Rousham et al. 2020). These figures show that there is moderate variation in consumption patterns. However, because no city‐specific estimates existed for Kumasi before fieldwork, a conservative assumption of *p* = 0.5 was adopted and used in Cochran's formula. This value provides the maximum possible variance and generates the highest possible sample size, hence the adequate statistical power, even in cases where the true population is low (Lohr 2021; Cochran 2009). This value maximizes sample size and ensures adequate statistical power, representing standard practice in exploratory social and nutrition research when baseline prevalence data are unavailable (Lohr 2021; Cochran 2009). The conservative approach provides the maximum possible variance and assists in obtaining wide coverage in terms of income and residential status within urban Ghana.

A margin of error of 0.08 was selected to balance statistical rigor with practical constraints common in urban surveys such as respondent availability and short time periods for data collection in densely populated areas. While no prior fruit and vegetable studies have used this exact margin, our approach aligns with established sampling principles for exploratory research in social science and consumer behavior, where margins of 5%–10% are standard when balancing precision with logistical challenges (Hasan and Kumar 2024; Lohr 2021; Cochran 2009).

To ensure that the sample reflected the socioeconomic diversity of Kumasi Metropolis, this study employed a multistage sampling technique validated in comparable urban food studies. The metropolis was stratified into low‐, middle‐, and high‐income categories (Table 1) based on existing socioeconomic classifications by Asiedu et al. (2024) and Nimoh et al. (2018), which have been shown to correlate with food purchasing behaviors in urban Ghanaian contexts (Edgmand 1987).

**TABLE 1:** Classification of Kumasi Metropolis communities by income level.

High income	Dadiesoaba, Asokwa, West Ayigya, Mbrom, Adiebeba, Adiembra, Ahodwo, Danyame, Odeneho Kwadaso, Aketego, Bomso, Bompe, Ridge, Nhyiaso, Extension, Parakuo Estate, Daban New Site, New Amakom Extension, Asokwa Residential Area
Middle income	Asafo, Amakom, Airport, Bantama, Dichemso, Aprade, New Tafo, Asebi, Anyinam, Kuwait, Atonsu, New Atonsu, Gyenyase, New Agogo, Adoato, Kyirapatre Estate, Bohyen, Adumanu, Adumanu Extension, Asanti Newtown, Apiri, North Suntreso, Kotei, South Suntreso, Boadi West Patase, Ohwimase, Kwadaso Estate, Santase Odumase Extension, Patase, Kentinkrono
Low income	Apatrapa, Dompoase, Aboabo, Moshie Zongo, Dichemso, Old Tafo, Ayigya Zongo, Dakwadwom, Sawaba, Yalwa, Daban, Kaase, Sokoban, Nsenee, Ahinsan, Anwomaso, Gyinyase, Adukrom, Asawase, Buobai, Nima, Pakuso, Abrepo, Sokoban, Amanfrom, Yenyawso, Buokrom, Ayeduase

Five communities per stratum were selected using random sampling. Within each selected community, systematic sampling (*k* = 5 interval) was applied from random starting points, with households screened for eligibility (the primary food buyer is present). The primary buyer (defined as responsible for ≥ 50% of household food procurement and verified during interviews) was surveyed to capture the decision‐making dynamics related to fruit and vegetable consumption. A total of 15 communities, five from each income group were sampled, with ten households selected per community (Table 2). This resulted in an overall final sample of 150 respondents. This design achieved a balanced representation across income groups while accommodating the logistical constraints of urban fieldwork. While the study employed equal sample sizes across income strata for analytical balance, this may not perfectly mirror the true population distribution. We acknowledged that this proportional imbalance could influence generalizability by slightly over‐ or under‐representing some income groups. However, the design was purposively chosen to allow robust comparison of dietary behavior across socioeconomic contexts.

**TABLE 2:** Selected communities and sample size.

Income category	Residential communities	Number of households
High income	West Ayigya, Asokwa Residential Area, Danyame, Odeneho Kwadaso, Nhyiaeso	10 each
Middle income	Bantama, Asafo, Amakom, New Tafo, Atonsu	10 each
Low income	Adukrom, Aboabo, Abrepo, Old Tafo, Suame	10 each

Prior to data collection, the questionnaire was pretested in a non‐sampled community to improve the quality of the instrument for clarity and consistency. The questionnaire consisted of three sections. The first section captured sociodemographic information, including age, gender, education level, occupation, household size, and income. The second section addressed consumption patterns, including the frequency of fruit and vegetable intake, purchasing behavior, and sources. The third section explores consumer preferences such as freshness, price, origin, and variety. Trained enumerators administered the questionnaires, and the responses were digitized and cleaned before analysis.

This study was reviewed and approved by the Research Ethics Committee of the Institute of Research, Innovation, and Development, Kumasi Technical University (Approval No. IRID/EC2025/HS0071). All participants provided informed consent prior to data collection, and participation was entirely voluntary. Respondents were informed of the study's purpose, assured of confidentiality, and permitted to withdraw at any stage without consequence. No identifying information was collected.

### 3.3 Method of Data Analysis

Descriptive statistics were used to analyze the socioeconomic characteristics and consumption patterns of the respondents. Following prior consumer behavior studies, such as Wongnaa et al. (2024), Ogunleke and Baiyegunhi (2019), and Ndenga et al. (2017), the Kendall's W was effectively used to analyze ranking‐based preferences for food product attributes. This non‐parametric statistical test measures the consensus among multiple raters when ranking a set of items (Asiedu et al. 2024). In this study, it was used to assess the consistency in how respondents prioritized intrinsic, extrinsic, and environmental attributes of fruits and vegetables. Kendall's W ranges from 0 (no agreement) to 1 (complete agreement). A higher W indicates a stronger consensus among the respondents. The test is particularly suitable when the data involves ordinal rankings, as in the case of consumer preferences. Kendall's W is calculated using the following formula:

(3) $$W=\frac{12S}{p^{2}\left(n^{3}-n\right)-\mathrm{pT}}$$

where *p* represents the number of attributes, n is the sample size, and T and S are the correlation factors for the bound ranks and sum of square statistics, respectively. The significance of Kendall's W is determined by testing the following hypothesis:

*There is no consensus among consumers regarding the ranking of attributes.*

*There is a consensus among consumers regarding the ranking of attributes.*

The decision to accept or reject the null hypothesis in Kendall's *W* analysis is based on comparing the calculated chi‐square value to its critical value. If the calculated chi‐square is greater than a critical value, the null hypothesis of no agreement is rejected for the alternative, indicating a statistically significant level of agreement among respondents on how they rank the attributes.

To measure the key attributes sought by consumers of FVs in this study, the respondents were asked to rank in order of importance the listed attributes on a scale of 1–8. The most important attribute was ranked as 1st and the least was ranked as 8th for both fruits and vegetables.

Consumers perceived benefits of vegetables and fruits were also analyzed using a 5‐point Likert scale: strongly disagree (1), disagree (2), neutral (3), agree (4), and strongly agree (5). The mean score was then computed to understand respondents' perceptions of the perceived benefits (nutrition, and health) of vegetables and fruits.

Additionally, to identify the significant factors influencing fruit and vegetable consumption patterns among urban consumers, the Negative Binomial Regression (NBR) using the NB2 (Negative Binomial type 2) formulation was employed. This is a robust generalization of Poisson regression that accounts for overdispersion through a quadratic variance function (Cameron and Trivedi 2013; Hilbe 2011). Given that the outcome variables, self‐reported weekly servings of fruits ((FVFruit) and vegetables (FVVeg), are non‐negative count data with evidence of overdispersion (variance > mean, see Appendix A; Table A1), conventional linear regression models such as Ordinary Least Squares (OLS) or Seemingly Unrelated Regression (SUR) are inappropriate. Instead, the general form of the Negative Binomial model was suitable which follows the NB2 specification, as presented in Pagui et al. (2022) and Cameron and Trivedi (2013), and is expressed as:

(4) $$E\left(\frac{Y_{i}}{X_{i}}\right)=\exp\left(\beta_{0}+\sum_{j=1}^{k}\beta_{j}X_{\mathrm{ij}}\right)$$

(5) $$\mathrm{Var}\left(Y_{i}|X_{i}\right)=\mu_{i}+\alpha \mu_{i}^{2}$$

where α>0 is the dispersion parameter that captures overdispersion, and $\mu_{i}$ is the conditional mean of the distribution. This formulation has been empirically validated as superior to Poisson regression in handling over dispersed count data in nutritional studies (Tiara et al. 2023).

The choice of NBR is supported by prior research on dietary behavior, where count‐based outcomes frequently exhibit overdispersion due to unobserved heterogeneity (Johnson et al. 2022; Deagle et al. 2019). Preliminary diagnostic tests, including Pearson's and Deviance goodness‐of‐fit statistics (*p* < 0.001), confirmed that the Poisson model was unsuitable, justifying the use of NBR (see Appendix A). Each dependent variable (FVFruit and FVVeg) was modeled separately with the same set of covariates to ensure comparability, following established econometric practices in nutrition studies (Mergenthaler et al. 2009). Applied to this study, the empirical specifications for the two dependent variables are as follows:

(6) $$\begin{array}{c} E\left(\frac{{\text{FVFruit}}_{i}}{X_{i}}\right)=\exp(\beta_{0}+\beta_{1}{\mathrm{Age}}_{i}+\beta_{2}{\text{Gender}}_{i}+\beta_{3}{\text{Education}}_{i}\\ +\beta_{4}{\text{MaritalStatus}}_{i}+\beta_{5}{\text{Dependents}}_{i}+\beta_{6}{\text{Income}}_{i}\\ +\beta_{7}{\text{Occupation}}_{i}+\beta_{7}{\text{Refrigerator}}_{i}+\beta_{8}{\text{HealthKnowledge}}_{i}\\ +\beta_{10}{\text{Accessibility}}_{i}+\beta_{11}{\text{Distance}}_{i}+\beta_{12}{\text{DecisionMaking}}_{i}\\ +\beta_{13}{\text{CulturalSocial}}_{i}+\alpha \end{array}$$

and

(7) $$\begin{array}{c} E\left(\frac{{\text{FVVeg}}_{i}}{X_{i}}\right)=\exp(\gamma_{0}+\gamma_{1}{\mathrm{Age}}_{i}+\gamma_{2}{\text{Gender}}_{i}+\gamma_{3}{\text{Education}}_{i}\\ +\gamma_{4}{\text{MaritalStatus}}_{i}+\gamma_{5}{\text{Dependents}}_{i}+\gamma_{6}{\text{Income}}_{i}+\gamma_{7}{\text{Occupation}}_{i}\\ +\gamma_{7}{\text{Refrigerator}}_{i}+\gamma_{8}{\text{HealthKnowledge}}_{i}+\gamma_{10}{\text{Accessibility}}_{i}\\ +\gamma_{11}{\text{Distance}}_{i}+\gamma_{12}{\text{DecisionMaking}}_{i}+\gamma_{13}{\text{CulturalSocial}}_{i}–\alpha \end{array}$$

where $X_{i}$ represents the vector of explanatory variables (see Table 3 for the full list).

**TABLE 3:** Description, measurement, and expected signs of variables in the SUR model.

Variable	Measurement	Expected sign	Related literature
**Dependent variable**			
${\text{FVFruit}}_{i}$	Number of fruit servings consumed per week		
$\text{FVVe}g_{i}$	Number of vegetable servings consumed per week		
**Explanatory variables**			
Age	Continuous variable: Number of years	+	Lima et al. (2023); Odum et al. (2018); Amo‐Adjei and Kumi‐Kyereme (2015); Dehghan et al. (2011)
Gender	Dummy variable: 1 = Male; 0 = Female	+/−	Lima et al. (2023); Marklinder et al. (2014); Dehghan et al. (2011)
Education	Dummy variable: 1 = if the highest education is high school or higher; 0 = if primary school education or less	+	Lima et al. (2023); Stadlmayr et al. (2023); Oppong‐Kyeremeh and Bannor (2021); Dehghan et al. (2011)
Marital status	Dummy variable: 1 = Married; 0 = otherwise	+	Dehghan et al. (2011); Pollard (2002)
Dependents	Continuous variable: Number of individuals financially dependent on respondent	+	Carroll et al. (2024); De Albuquerque and Albala (2021)
Income	Continuous variable: Monthly household income measured in Ghana cedi (GH₵)	+	Dijkxhoorn et al. (2021); Mensah et al. (2021); Frank et al. (2019); Meng et al. (2018)
Occupation	Dummy variable: 1 = if respondent is employed; 0 = unemployed	+	Lima et al. (2023); Amo‐Adjei and Kumi‐Kyereme (2015); Dehghan et al. (2011)
Refrigerator	Dummy variable: 1 = if respondent owns a refrigerator; 0 = otherwise	+	Marklinder et al. (2014)
Nutrition knowledge	Dummy variable: 1 = if respondent is very or somewhat knowledgeable about nutritional benefits of FV; 0 = otherwise	+	Arbianingsih et al. (2021); Oppong‐Kyeremeh and Bannor (2021)
Accessibility	Dummy variable: 1 = if respondent reports fresh FV as easy or readily accessible; 0 = otherwise	+	Okekunle et al. (2024); Wind et al. (2006)
Distance	Continuous variable: Measured in kilometers from home to usual FV purchase location	−	Sinyolo et al. (2020)
Household decision making	Dummy variable: 1 if self‐decision making; 0 = otherwise	+	Arbianingsih et al. (2021)
Cultural social event	Dummy variable: 1 = if respondent reports social or cultural events in community that promote FV; 0 = No	+	Kähkönen et al. (2021); Oppong‐Kyeremeh and Bannor (2021); Helsel et al. (2019)

Accessibility was both subjective and objective, which measured various levels of access to fruits and vegetables by households. The subjective measure provided questions to the respondents on whether they perceived fruits and vegetables as easy or not easy to access in their neighborhood in terms of perceived availability, convenience, and affordability. To complement this, the objective indicator, distance (in kilometers) between the homes of the respondents and their frequent fruit and vegetable purchase location was also added as an independent covariate in the model. This measure was used as a proxy for physical accessibility. These two variables together offer a more realistic evaluation of access conditions in the Kumasi Metropolis where convenience and physical proximity can vary because of the cost of transport, market congestion, and infrastructural condition of the roads. Robust standard errors were applied to correct for heteroskedasticity, and incidence rate ratios (IRRs) were reported for interpretability.

In this study, the reported marginal effects are Average Marginal Effects (AMEs), which represent the mean of the individual marginal effects (MEs) across all respondents. This provides an interpretable measure of how each explanatory variable affects the average number of weekly fruit or vegetable servings, holding other factors constant. The method for calculating AMEs is explained in Appendix C.

Marginal effects (ME) were calculated as average marginal effects (AME) across all observations, representing the mean change in the number of expected servings of fruit or vegetables in response to a one‐unit change in each of the explanatory variables, all things being equal. This approach aligns with methodological recommendations for count data analysis (Cameron and Trivedi 2013) and has been validated in similar studies on food consumption (Damari and Kissinger 2018; Zezza et al. 2017). By addressing overdispersion and heteroskedasticity, the NBR provides reliable coefficient estimates, enhancing the policy relevance of our findings. To ensure the reliability of the parameter estimates, multicollinearity diagnostics (variance Inflation Factors (VIF)) were computed for all explanatory variables. As both models used identical predictors, the same VIF results apply, with all values below the conventional threshold of 10, indicating no multicollinearity (Gujarati and Porter 2009). Detailed results are provided in Appendix B, Table A2.

## 4 Results and Discussion

### 4.1 Socio‐Economic Characteristics of Respondents

The socio‐economic and demographic characteristics of the respondents are presented in Table 4. The majority of the respondents in this study were males (55%), most were unmarried (54%), with an average of 33 years and had attained a higher level of education where approximately 92% had secondary education and above. The sample is reflective of a typical urban working‐class population where young male adults make independent dietary choices (Amo‐Adjei and Kumi‐Kyereme 2015). The higher level of educational attainment suggests a population that can make informed dietary choices and consumption preferences. Both genders participate in fruit and vegetable (FV) purchasing decisions on an almost equal basis in urban areas of Kumasi, which is contrary to previous studies where women constitute the main decision makers. (Silva et al. 2023; Nicklett and Kadell 2013).

**TABLE 4:** Socio‐demographic characteristics and dietary patterns of respondents (*n* = 150).

Variable	Category/Statistic	Frequency	Percentage
Gender	Male	83	55.3
	Female	67	44.7
Marital status	Single	81	54.0
	Married	52	34.7
	Divorced	17	11.3
Educational background	Illiterate	9	6.0
	Primary	2	1.3
	Secondary	36	24.0
	Vocational/Technical	48	32.0
	Tertiary	50	33.3
	Post‐graduate	5	3.4
Household role	Household Head (Breadwinner)	49	32.6
	Co‐breadwinner	46	30.7
	Dependent	46	30.7
	Independent (No obligation)	9	6.0
Occupation	Unemployed	48	32.0
	Self‐employed (Informal)	47	31.3
	Employed (Private sector)	33	22.0
	Employed (Public/government)	22	14.7
Monthly income (GH¢)	500–1000	62	41.3
	1001–2000	17	11.3
	2001–3000	16	10.7
	3001–4000	14	9.3
	4001–5000	11	7.4
	5000+	30	20.0

Continuous variable	Mean (SD)	Minimum	Maximum
Age	33.14 (9.89)	17	71
Number of dependents	2.37 (1.89)	1	8
Weekly fruit servings	5.77 (10.00)	0	56
Weekly vegetable servings	5.91 (9.38)	0	64
Weekly serving of FV consumed	11.67 (18.49)	0	120

*Note:* Standard deviations are in parentheses.

Data from the study shows that approximately 63% of respondents were identified as either breadwinners or co‐breadwinners, while 31% were dependents. As expected, there were income disparities among the sample where approximately 53% earned ≤ GH₵2000 (US$ 450) monthly while 20% earned > GH₵5000 (US$ 890), and this can potentially shape consumption patterns (Pechey and Monsivais 2016). Indeed, as shown in Table 4, on average, respondents consumed 5.77 fruit and 5.91 vegetable servings weekly, for a total of 11.67 servings, which is far below the WHO recommendation of 35 servings/week. The variability in consumption, as indicated by the high standard deviations for both fruit and vegetable intake (10.00 and 9.38, respectively), suggests that access to these FVs varies significantly among the urban population.

### 4.2 Preferred Attributes Influencing Fruit and Vegetable Purchases in Urban Areas

A summary of consumer choice and preference when purchasing fruits and vegetables (FV) is presented in Table 5. Three main consumer attributes were analyzed, that is, the intrinsic, extrinsic, and environmental dimensions.

**TABLE 5:** Preferred attributes of fruits and vegetables among urban consumers.

Attribute category	Preferred attribute	Mean	Rank
Intrinsic	Taste	2.99	1st
	Appearance	4.02	2nd
	Freshness	4.29	3rd
	Color/brightness	4.57	4th
	Wholesomeness‐no bruises, no cuts	4.84	5th
	Nutritional value	4.89	6th
	Firmness	5.08	7th
	Flavor/smell	5.31	8th
Extrinsic	Price	2.31	1st
	Nutritional value	2.65	2nd
	Health benefits	2.99	3rd
	Source/origin	4.92	4th
	Brand	5.27	5th
	Labelling	5.70	6th
	Package	5.78	7th
	Seller/marketplace	6.39	8th
Environmental	Organically grown type	3.21	1st
	Pesticide effect on fruit and vegetables	3.50	2nd
	Seasonality of fruit and vegetables	3.82	3rd
	Dirt or debris	4.07	4th
	Contamination from water source	4.63	5th
	Certified product	4.93	6th
	Labelling	5.82	7th
	Conventionally grown type	5.98	8th

Test statistics	Intrinsic	Extrinsic	Environmental
Kendall's W	0.091	0.427	0.178
Chi‐Square	95.149	448.702	186.553
df	7	7	7
*p*	0.000	0.000	0.000
Observations	150	150	150

*Note:* Lower mean values indicate higher preference ranking.

Results from the study show that the important intrinsic attributes that influenced choice of FV by consumers were taste (mean = 2.99), followed by appearance (mean = 4.02) and freshness (mean = 4.29). This highlights a clear tendency among consumers to prioritize sensory qualities, with taste leading to their purchasing decisions. This result is consistent with the findings of Török et al. (2023) and Dana et al. (2021), that emphasized the primacy of intrinsic attributes (taste) in shaping food preferences, particularly in urban environments, where sensory appeal may outweigh considerations such as nutrition. Similarly, Cardona et al. (2023) opined that taste and appearance consistently rank as top priorities for consumers, even when other factors are considered.

Regarding extrinsic attributes, the leading considerations as ranked by consumers were the price (mean = 2.31), nutritional value (mean = 2.65), and health (mean = 4.92) benefits of FV. These results clearly indicate that cost remains a major concern in FV consumption, highlighting the economic constraints faced by urban consumers. This observation supports the earlier work of Stadlmayr et al. (2023), who found that affordability significantly affects food purchasing patterns. Consumer choice and preference are therefore subject to price as one important consideration.

On the environmental attributes, the respondent's preference was informed by whether the FV was organic (mean = 3.21), the effect of pesticide on the fresh FV (mean = 3.50) and seasonality of FV (mean = 3.82) (see Table 5). Organic FV are regarded as safe and high value FV by health‐conscious consumers (Wongnaa et al. 2024), hence the high preference and ranking for that attribute. This finding suggests that consumers are aware and thus pay attention to their health and environmental factors that have implications for food choices. In contrast, attributes such as certified products, labelling, and conventionally grown types of FV ranked lowest. A plausible reason for this finding is that the formal indicators of quality common in developed markets in, say, the E.U and North America, have not yet gained substantial attention in the study area. Rana and Paul (2017) and Ochieng et al. (2018) reported similar gaps in markets where certification and branding are not primary drivers of purchasing decision and choice of consumers.

The test statistics for the degree of agreement using Kendall's W were 0.091, 0.427, and 0.178 for intrinsic, extrinsic, and environmental attributes, respectively. These values indicate low agreement for intrinsic, moderate agreement for environmental, and relatively strong consensus for extrinsic attributes. The null hypothesis of no agreement among respondents is therefore rejected in favor of the alternative, as the chi‐square values for all three groups were statistically significant (*p* < 0.000). Therefore, it can be concluded that, despite differences in individual views, respondents showed statistically significant agreement on how they ranked the attributes that guide their fruit and vegetable purchasing decisions.

### 4.3 Perceived Benefits and Importance of FV Consumption

The second objective of this study was to assess consumer‐perceived benefits and importance of fruit and vegetable (FV) consumption, focusing specifically on nutrition, and health dimensions. Consumers ranked their perceived benefits of FV consumption based on 8‐item construct on a scale of ‘strongly agree’ (1) to ‘strongly disagree’ (5). As shown in Table 6, the findings present a clear picture, with mean scores, of how urban consumers in the study area interpret the role of FV in their diets. Results showed that the most important perceived benefits of FV consumption were for good health (mean = 3.34), balanced diet (mean = 3.30), and good nutritional source (mean = 3.29). Consumers thus exhibit positive tendency for FV on account of the health and nutritional benefits to be derived. A good understanding and positive mindset of respondents about the benefits of FV consumption is critical to promoting overall health. This finding aligns with those of previous studies that reported priority of consumers for health benefits of FV (Wongnaa et al. 2024). Importantly, this finding is significant as, it resonates with Ghana's Ministry of Health campaign to promote consumption of fruits and vegetables as a healthy lifestyle for the citizenry.

**TABLE 6:** Perceived benefits of fruit and vegetable consumption among urban consumers.

Consumer perceived benefit constructs	Strongly disagree (1)	Disagree (2)	Fair (3)	Agree (4)	Strongly agree (5)	Mean score
FVs are important for health benefits	12	27	32	56	23	3.34
FVs are essential for balanced diet	13	27	28	66	16	3.30
FVs are essential for good nutrition	18	18	37	57	20	3.29
FVs prevents diseases (preventive)	13	25	41	57	14	3.23
FVs consumption is important only for sick or unhealthy people	22	33	32	38	25	3.07
FVs is important for only vegetarians	23	28	40	38	21	3.04
FVs must be consumed every day	20	31	46	36	17	2.99
FVs consumption is important only for children	24	35	31	40	20	2.98

Despite the perceived high value or benefits of FV, daily consumption was rated very low (mean = 2.99) by the respondents, indicating a disconnect between awareness and practice or routine behavior. Additionally, the respondents moderately endorsed statements based on some assumptions and beliefs that, for example, that ‘FV are essential for people who are sick’ (mean = 3.07), and that ‘FV are for vegetarians’ (mean = 3.04), while the ‘benefits of FV to children’ were weakly scored (mean = 2.98). These moderate to weak mean scores highlight lingering misperceptions that may weaken the perceived universal relevance of FV in everyday diets and particularly for children. The causal nature of FV consumption and these misconceptions probably account for the disconnect and low rates of consumption.

These findings align with SDG 3 targets for promoting healthy lives (WHO 2018), particularly in urban African contexts, where nutrition security requires coordinated policy interventions to address affordability (Herforth and Ahmed 2015), accessibility (Okekunle et al. 2024), and dietary misconceptions (Stadlmayr et al. 2023).

### 4.4 Urban Consumption Patterns of Fruits and Vegetables

**FIGURE 1:**
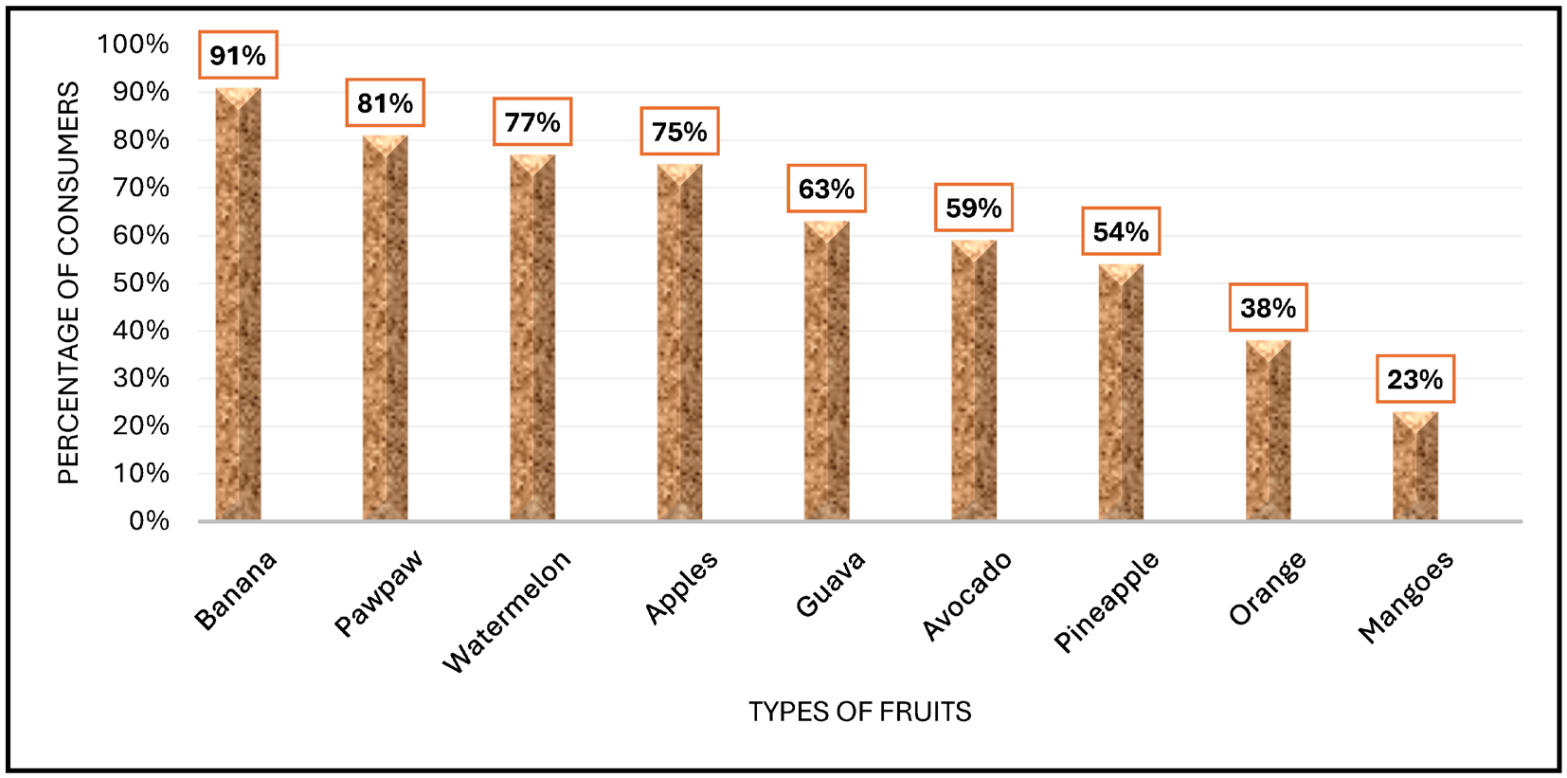
Proportion of respondents and preferred fruits. *Source:* Primary survey data, 2024.

**FIGURE 2:**
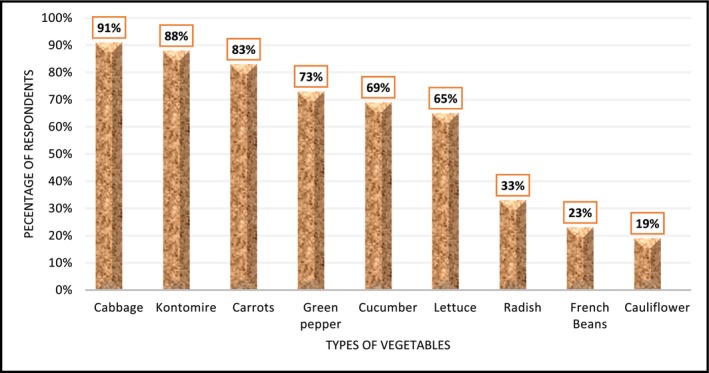
Proportion of respondents and preferred vegetables. *Source:* Primary survey data, 2024.

**FIGURE 3:**
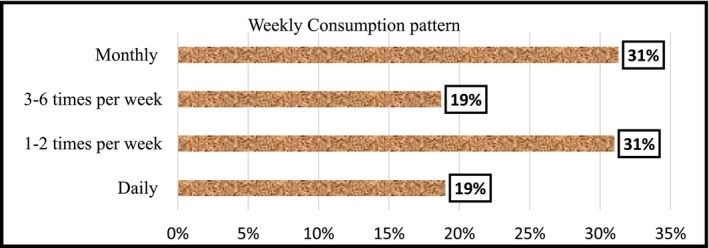
Weekly consumption patterns of fruits and vegetables. *Source:* Primary survey data, 2024.

**FIGURE 4:**
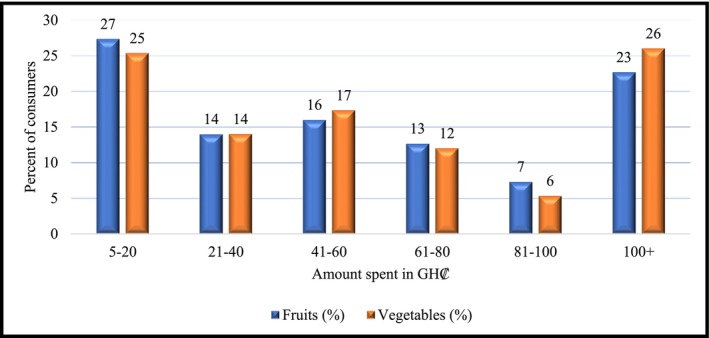
Weekly household expenditure (GH¢) on fruits and vegetables. *Source:* Primary survey data, 2024.

Consumption patterns of fruits and vegetables were described based on the preferred choice, frequency of consumption, source of FV and expenditure. The results are presented in Figures 1, 2, 3, 4 and Table 7. Figure 1 shows that the fruits preferred by most consumers were bananas (about 91% of respondents), pawpaw (81%), watermelons (77%), and apples (71%), while the least preferred were mangoes (23% of respondents). The result shows that fruit consumption of preference was relatively higher or common.

**TABLE 7:** Source of purchase of fruits and vegetables by urban consumers.

Sources of purchase	Frequency	Percentage
Farm gate or Home garden	15	10.0
Market‐local or wet market	60	40.0
Wayside/street vendors	46	30.7
Supermarket/grocery shop	29	19.3

Figure 2 shows the proportion of consumers indicating the preferred vegetables they patronize. The consumers preferred mostly green leafy vegetables such as cabbage (91%) and cocoyam leaves (kontomire) (87%), followed by carrots (83%), whilst the least preferred were cantaloupe (11%) and cauliflower (19%). While no specific reasons were assigned for choices made, key factors that drive preference for vegetable choice can include seasonal availability, cost, taste, or other factors. This echoes previous observations by Keding et al. (2017) and Ruel et al. (2005) who posit that affordability and cultural familiarity shape dietary choices.

The frequency at which consumers take in FVs constitutes an important indicator of consumption pattern. As shown in Figure 3, only 19.30% of the respondents reported daily FV consumption, while approximately 31% consumed it once or twice a week, and the same percentage consumed it less frequently (monthly). Though the data could not capture the exact quantities consumed, the relatively small proportion of respondents who reported daily consumption of FVs is an indication of acutely low daily intake compared to WHOs recommendations. The inconsistent intake of FVs as depicted in Figure 3, despite the general availability of these foods, indicates a disconnect between accessibility and routine dietary behaviors. The erratic consumption of FVs by the majority of respondents could be attributed to the casual approach to dietary concerns by consumers in SSA where average diets are largely carbohydrate dense. Ruel et al. (2005) found that income and supply chain constraints within the urban food systems discourage regular consumption of FVs in most of sub‐Saharan African cities.

In terms of sourcing behavior, the data presented in Table 7 showed that most respondents relied on the informal markets to purchase FVs. Marketing and sale of agri‐food products including FVs in Ghana occur predominantly on the informal market (Kushitor et al. 2023). It is therefore not surprising that 40% and about 31% of respondents relied on local or wet markets and street vendors respectively to purchase FVs. Supermarkets were the third choice of market outlet or source for consumers (about 19%) while only 10% of respondents sourced FVs from the farmgate or home gardens. The results further highlight the dominance of informal or wet markets such as mainstream market outlets and supply channels even in typical urban food systems more than formal and high‐end outlets.

Next, the respondents were asked to indicate estimates of expenditure made on FV on a weekly basis. The results presented in Figure 4 shows that approximately 27% and 25% of respondents spent only GH₵5–20 weekly on fruits, and vegetables respectively. A similar proportion of respondents spent more than GH₵100 weekly on FVs (see Figure 4), suggesting both affordability constraints and income‐driven disparities. The results are consistent with those of Drewnowski et al. (2020) and Herforth and Ahmed (2015), who reported that economic access or income stratifies healthy food consumption.

### 4.5 Determinants of Urban Fruit and Vegetable Consumption

The factors influencing fruit and vegetable consumption patterns among urban household consumers are presented in Table 8. The diagnostic tests validated the robustness of the Negative Binomial Regression (NBR) models for the analysis of fruit and vegetable consumption patterns. The highly significant likelihood‐ratio (LR) chi‐square statistics (100.22 for fruits and 97.83 for vegetables, both with *p* < 0.000) suggest that these models are well supported by the data, since the explanatory variables used in the model are adequately and strongly predictive. Therefore, the hypothesis that all coefficients are zero is decisively rejected (Cameron and Trivedi 2013). While pseudo‐*R* ^2^ values were examined (0.118 for fruits and 0.114 for vegetables), it should be emphasized that likelihood ratio tests, information criteria, and overdispersion parameters provide more reliable evidence of model fit in count data regression (Hilbe 2011).

**TABLE 8:** Negative binomial regression results for urban fruit and vegetable consumption.

Variable	Fruit model			Vegetable model		
	Coef.	IRR	ME	Coef.	IRR	ME
Age	−0.014 (0.012)	0.986	−0.078	−0.015 (0.011)	0.985	−0.086
Gender	0.182 (0.158)	1.199	1.018	0.036 (0.146)	1.036	0.206
Education	0.108 (0.336)	1.114	0.604	0.193 (0.302)	1.213	1.114
Marital status	0.190 (0.186)	1.209	1.065	−0.164 (0.172)	0.849	−0.945
Dependents	0.073 (0.065)	1.076	0.409	0.149 (0.055)***	1.161	0.862
Income	−0.008 (0.006)	1.000	−0.004	−0.002 (0.001)	0.999	−0.001
Occupation	0.025 (0.226)	1.025	0.140	0.203 (0.207)	1.225	1.173
Refrigerator ownership	−0.318 (0.221)	0.728	−1.782	−0.553 (0.205)***	0.575	−3.191
Nutrition knowledge	0.575 (0.217)***	1.778	3.225	0.208 (0.194)	1.231	1.200
Accessibility	1.097 (0.199)***	2.995	6.149	1.162 (0.180)***	3.196	6.704
Distance	0.004 (0.002)**	1.000	0.020	0.005 (0.002)***	1.005	0.027
Household decision‐making	−0.183 (0.193)	0.832	−1.028	−0.362 (0.174)**	0.696	−2.088
Cultural/social influence	0.500 (0.187)***	1.649	2.803	0.346 (0.170)**	1.414	1.997
Constant	0.688 (0.588)	1.951		0.991 (0.513)	2.694	
LR Chi^2^ (13)	100.22			97.83		
*p* > Chi^2^	0.000			0.000		
Pseudo *R* ^2^	0.118			0.114		
Log‐Likelihood	−374.872			−379.262		
Overdispersion	0.537			0.441		
LR test of alpha = 0 (chibar^2^)	457.22***			26.42***		
AIC crit. (AIC)	779.744			788.524		
BIC crit. (BIC)	824.904			833.684		
Observations	150			150		

*Note:* Standard errors are in parentheses. IRR is incident rate ratio. ME is average marginal effects (AME) computed across all observations, representing the mean change in expected weekly fruit or vegetable servings for a one‐unit change in each explanatory variable. ME is marginal effects. ***1%, **5%, *10%.

It is particularly important to the choice of model that the significant log‐likelihood (LR) tests of alpha (fruits: 457.22, vegetables: 26.42) confirm that there is substantial overdispersion in the count data (*α* = 0.537 and 0.441, respectively), which justifies the adoption of NBR instead of Poisson regression. The Akaike and Bayesian information criteria (AIC/BIC) further supported the NBR as the best‐fitting model to accurately describe the observed data. To interpret the estimates meaningfully, the incidence rate ratio (IRR) and marginal effects (ME) were computed.

The findings indicate that the most consistent predictors of fruit and vegetable consumption patterns are the number of dependents, refrigerator ownership, accessibility, nutrition knowledge, distance, household decision making, and socio‐cultural factors.

Individuals in households with improved physical access to fresh produce reported significantly higher consumption of both fruits and vegetables, with incidence rate ratios (IRRs) of 2.995 and 3.196, respectively. This indicates that such individuals were roughly three times more likely to consume fruits and vegetables frequently than those with limited access. On average, this translates to approximately six additional servings per week, highlighting the strong association between accessibility and dietary behaviors. These findings align with previous studies, where Okekunle et al. (2024) and Wind et al. (2006) reported increases in fruit and vegetable consumption that were associated with geographic and economic proximity to the location, specifically in urban areas, where transportation and storage issues are the most critical. Within the framework of the Theory of Planned Behavior (Ajzen 1991), this relationship reflects perceived behavioral control. Improved access may be associated with fewer physical and psychological barriers to action and greater confidence in individuals' abilities to perform the intended behavior. This result aligns with the view that behavioral control, when supported by favorable conditions, is associated with a greater likelihood of translating positive intentions into actual consumption behavior. Similarly, the significant association between nutritional knowledge and fruit consumption (IRR = 1.778; ME = 3.225) further underscores the importance of attitudinal factors in the TPB. Knowledgeable individuals tend to have more favorable evaluations of healthy eating, which are associated with more favorable behavioral intentions. Similar observations were reported by Arbianingsih et al. (2021) and Sheline et al. (2024), who found that nutritional literacy enhanced positive attitudes toward fruit consumption. Although a similar trend was observed for vegetables (IRR = 1.231), it was not statistically significant. This discrepancy may suggest that health‐related attitudes toward fruits are more strongly internalized than those toward vegetables, possibly due to perceived differences in taste, convenience, or health issues. These results are consistent with those of Sniehotta et al. (2014), who found that cognitive awareness alone may not always translate into uniform behavioral responses. This finding suggests a need for targeted education to better communicate the nutritional importance of vegetables.

Cultural and social influences were also significantly associated with consumption patterns, increasing fruit and vegetable servings by 1% and 5%, respectively. Individuals actively engaged in cultural or community events were approximately 1.6 times more likely to consume fruits and 1.4 times more likely to consume vegetables at higher frequencies than non‐engaged households. This is consistent with the findings of Kähkönen et al. (2021) and Helsel et al. (2019), who emphasized the role of social norms and cultural contexts being associated with variations in dietary behaviors. Within TPB, these outcomes align with subjective norms, reflecting the perceived social pressure to conform to group expectations. In communities where social gatherings and cultural events emphasize shared meals, the consumption of fruits and vegetables may symbolize not only health consciousness but also social belonging. The results are in good agreement with earlier studies showing that collective social practices can reinforce habitual healthy eating (Bisogni et al. 2012). Thus, culturally embedded interventions, such as nutrition education linked to festivals or communal events, may be more closely associated with sustained fruit and vegetable intakes than purely individual‐based programs. Surprisingly, refrigerator ownership was negatively associated with fruit and vegetable consumption, with households owning refrigerators being 0.56 times less likely to consume vegetables and 0.73 times less likely to consume fruits. One possible interpretation is behavioral substitution, where individuals or households with refrigeration facilities may rely more on convenient or processed food. This explanation remains speculative, as the quantitative design of this study did not allow for the direct exploration of underlying behavioral motives. However, similar associations have been reported by Zheng et al. (2025) and Heard et al. (2020), who observed associations between refrigerator ownership and higher energy intake and dietary shifts toward energy‐dense and animal‐based foods. Likewise, Martinez et al. (2021) and Huang and Ren (2025) found that access to refrigeration was associated with increased consumption of processed and refrigerated products and changes in food storage practices. Within the TPB framework, this pattern may reflect perceived behavioral control, where increased storage convenience is associated with a preference for easy‐to‐store foods rather than fresh produce. Consequently, even when individuals or households have positive attitudes toward healthy eating, convenience‐related factors may weaken the translation of intentions into actual behavior.

Household decision‐making was significantly associated with vegetable intake (IRR = 0.696). Individuals who were not the sole decision‐makers reported significantly lower vegetable consumption, suggesting that shared or limited decision‐making may be linked to reduced autonomy in food choices. This finding is consistent with the TPB construct of subjective norms, where the expectations and influence of significant others such as spouses, elders, or cohabitants affect individual food‐related behaviors. Arbianingsih et al. (2021) reported similar patterns, noting that shared or subordinate decision‐making limits personal autonomy in food purchasing and preparation processes. The implication here is that, although causal relationships cannot be inferred from the current data, interventions aimed at promoting adequate vegetable consumption may benefit from incorporating gender‐sensitive and empowerment‐based approaches that support greater participation and autonomy in household food decision making. As anticipated, distance to the nearest fruit or vegetable source, despite small effect sizes, showed positive and significant relationships with both fruits and vegetables. This result may appear counterintuitive, but it can be understood through the lens of TPB, which posits that individuals with strong attitudes and intentions toward healthy eating are more likely to overcome environmental barriers. This suggests that distance becomes a less significant deterrent for individuals or households with firm intentions to maintain healthy eating habits. This finding supports similar findings reported by Rahkovsky and Snyder (2015) and Thornton et al. (2012) but contradicts those of Aggarwal et al. (2014) and Sharkey et al. (2010), who found fruit and vegetable consumption was not associated with distance but with supermarket choice.

Among the sociodemographic variables, the number of dependents was significantly associated with higher vegetable consumption (IRR = 1.161; *p* < 0.01) but not with fruit intake. This may reflect the perception of vegetables as essential, versatile, and cost‐effective foods suitable for household meals, particularly when compared with fruits. This interpretation aligns with the TPB, as caregiving responsibilities often shape food choices through attitudinal and normative pathways. These findings are consistent with previous studies that highlight how caregivers' nutritional attitudes and social expectations are associated with household dietary choices (Monterrosa et al. 2020; Christian et al. 2016). Other variables such as age, gender, income, education, marital status, and occupation exhibited no statistically significant influence on fruit or vegetable consumption. While the direction of these coefficients was generally consistent with expectations (e.g., positive signs for education and occupation), their lack of statistical significance suggests that, in this urban context, structural and behavioral factors may play a larger role than individual sociodemographic factors in shaping dietary patterns. These findings contrast somewhat with literature, such as Lima et al. (2023) and Dehghan et al. (2011), who found education and income to be significant predictors in more heterogeneous or rural populations. This reflects a leveling effect in urban areas, where shared infrastructure, access, and exposure to nutritional information reduce disparities linked to socioeconomic factors.

## 5 Conclusion and Policy Recommendations

This paper explored fruit and vegetable (FV) consumption patterns among urban consumers in Kumasi, Ghana, focusing on preferred attributes, perceived health benefits, actual consumption behavior, and determinants of consumption. The findings show that urban consumers tend to prioritize intrinsic attributes (taste, freshness, and appearance), extrinsic attributes (price and nutritional value), and environmental related attributes (organic and the presence of pesticides). The findings revealed a clear disconnect between awareness and practice, in that although most respondents valued the benefits of FV, daily consumption was not a priority. The relatively low agreement with statements supporting routine intake alongside lingering misconceptions that FV are mainly for children, the sick, or vegetarians suggests that these benefits are viewed as relevant only under specific conditions. Thus, both structural and informational barriers against FV consumption exist, especially those related to affordability and access. Consumers rely mostly on informal traditional market sources to purchase fruits and vegetables. The Negative Binomial Regression results indicated that number of dependents, accessibility, nutrition knowledge, cultural and social influences, and distance to markets were significantly associated with urban fruit and vegetable consumption. These relationships reflect the combined effects of attitudinal, normative, and perceived behavioral control components of the Theory of Planned Behavior. Accessibility and nutrition knowledge were positively associated with consumption, suggesting stronger perceived control and more favorable attitudes toward healthy eating, while cultural and social participation corresponded with subjective norms that support fruit and vegetable intake. In contrast, refrigerator ownership and household decision‐making were negatively related to vegetable consumption, possibly indicating behavioral substitution toward convenient or processed foods and normative factors that hinder the translation of intention into practice. Among the sociodemographic variables, only the number of dependents showed a significant positive association with vegetable intake, which is consistent with caregiving‐driven food choices. These findings show that behavioral and contextual factors, rather than economic attributes, serve as the primary determinants of urban dietary behavior.

This paper makes the following contributions to increase fruit and vegetable (FV) consumption in urban Ghana. First, the study recommends that key stakeholders, including the government agency (Ministry of Health) and nutritionists, must pursue aggressive educational campaigns that sensitize the citizenry about the nutritional, environmental, and long‐term health benefits of FV consumption. To be effective, campaigns must emphasize the necessity of daily intake for all demographic groups and should be embedded in culturally resonant formats, including community events, markets, and faith‐based gatherings. Engaging trusted cultural networks such as community groups, market associations, and faith‐based organizations as nutrition advocates or ambassadors could enhance the sustainability and acceptance of dietary interventions. Consumers demand fresh FVs at the right place and at an affordable price, and these are key to meeting daily dietary requirements. In addition, the main agents across the urban FV supply chain, especially informal vendors and small retailers, should be encouraged to capitalize on consumers' preference for intrinsic qualities such as taste, appearance, and freshness. Finally, given that household decision‐making was negatively associated with vegetable consumption, nutrition interventions should be intentionally designed to empower caregivers (often women) and promote shared food decision‐making, particularly in settings where gender roles or economic dynamics may limit food choice autonomy. Culturally tailored programs could leverage maternal roles in food provisioning while educating male household heads on FV benefit.

## 6 Limitations

Like all empirical studies, this study has some limitations. First, the study relied on cross‐sectional data from a single urban metropolis, Kumasi, which limits causal inference and generalizability to peri‐urban or rural settings where socioeconomic conditions and food environments differ. Future studies should assess whether the observed relationships, such as those between accessibility, knowledge, and consumption, persist across different contexts and over time. Second, accessibility was measured using perceived and distance‐based indicators, which may not fully capture the complexity of market density or travel time. Incorporating geospatial data could provide more objective measures in future studies. Third, fruit and vegetable intakes were self‐reported, raising the possibility of recall or social desirability bias. Complementary methods such as dietary recalls or food diaries could improve data accuracy. Fourth, the study did not control for seasonality, which may influence the availability and consumption of fresh produce in Ghana. Finally, while the present design identified behavioral patterns, qualitative or mixed‐method approaches could deepen the understanding of underlying motivations, particularly the observed behavioral substitution linked to refrigerator ownership. Despite these limitations, our findings provide valuable evidence of associations in urban dietary behavior that warrant further investigation through longitudinal or experimental studies.

## Author Contributions

**Stephen Opoku‐Mensah:** conceptualization, methodology, writing – original draft, writing – review and editing, formal analysis, visualization. **Collins Yeboah Asiedu:** investigation, methodology, writing – review and editing, formal analysis, data curation. **Benjamin Sarfo:** investigation, validation, writing – review and editing, visualization.

## Data Availability

The data that supports the findings of this study are available from the corresponding author upon reasonable request.

## Appendix A

**TABLE A1:** Goodness‐of‐fit tests for negative binomial regression models.

Statistic	Fruit model	Vegetable model	*p*
Deviance goodness‐of‐fit	757.73	621.39	0.000
Pearson goodness‐of‐fit	972.49	769.07	0.000
Degrees of freedom (df)	136	136	—

*Note:* Both models reject the null hypothesis (perfect fit) at *p* < 0.001, confirming overdispersion and justifying the use of Negative Binomial Regression.

## Appendix B

**TABLE A2:** Multicollinearity diagnostics (variance inflation factors).

Variable	VIF	Tolerance (1/VIF)
Age	2.76	0.36
Gender	2.67	0.37
Education	1.47	0.68
Marital status	1.51	0.66
Dependents	2.32	0.43
Income	2.67	0.37
Occupation	2.08	0.48
Refrigerator ownership	1.86	0.54
Nutrition knowledge	1.44	0.69
Accessibility	1.50	0.67
Distance	1.12	0.90
Household decision‐making	1.36	0.73
Cultural/social influence	1.43	0.70
Mean VIF	1.74	

## Appendix C

### Computation of Average Marginal Effects (AME)

The Average Marginal Effects (AMEs) were calculated following the post‐estimation of the Negative Binomial Regression (NBR) model. This analysis helps understand how the explanatory variables influence weekly fruit and vegetable intake. For each explanatory variable $x_{k}$, the marginal effect on the expected count $E\left(y_{i}|X_{i}\right)$ is expressed as:

(A1)
$$\frac{\partial E\left(y_{i}|X_{i}\right)}{\partial x_{k}}=B_{k}\times \exp\left(X_{i}\beta \right)$$

For binary variables, the marginal effect represents the discrete change in the predicted number of servings when $x_{k}$ changes from 0 to 1:

(A2)
$$∆E\left(y_{i}|X_{i}\right)=E\left(y_{i}|x_{k}=1,X_{-k}\right)-E\left(y_{i}|x_{k}=0,X_{-k}\right)$$

The Average Marginal Effect (AME) for each explanatory variable is then obtained by averaging the individual marginal effects across all n observations:

(A3)
$${\mathrm{AME}}_{k}=\frac{1}{n}\;\sum_{i=1}^{n}\frac{\partial E\left(y_{i}|X_{i}\right)}{\partial x_{k}}$$

These computations were done using the “*margins*” command in STATA 17 after estimating the NBR model with robust standard errors, consistent with established procedures for estimating marginal effects in count models (Cameron and Trivedi 2013; Williams 2012; Hilbe 2011). This method ensures that the reported marginal effect values accurately reflect the estimation process. The AMEs reported in Table 8 represent the average effect across all sampled respondents, making it easier to interpret the influence (effect size) of each predictor (continuous and binary) on weekly fruit and vegetable consumption.
